# Influence of Cytokines on Inflammatory Eye Diseases: A Citation Network Study

**DOI:** 10.3390/jcm11030661

**Published:** 2022-01-27

**Authors:** Beatriz G. Gálvez, Clara Martinez-Perez, Cesar Villa-Collar, Cristina Alvarez-Peregrina, Miguel Ángel Sánchez-Tena

**Affiliations:** 1 Faculty of Experimental Sciences, Universidad Francisco de Vitoria, 28223 Madrid, Spain; beatriz.ggalvez@ufv.es; 2 ISEC LISBOA—Instituto Superior de Educação e Ciências, 1750-179 Lisbon, Portugal; clara.perez@iseclisboa.pt (C.M.-P.); masancheztena@ucm.es (M.Á.S.-T.); 3 Faculty of Biomedical and Health Science, Universidad Europea de Madrid, 28670 Madrid, Spain; villacollarc@gmail.com; 4 Department of Optometry and Vision, Faculty of Optics and Optometry, Universidad Complutense de Madrid, 28037 Madrid, Spain

**Keywords:** cytokines, inflammation, citation network, eye, bibliometric

## Abstract

Background: The main objective of this study was to use citation networks to analyze the relationship between different publications on the impact of cytokines at an ocular level and their authors. Furthermore, the different research areas will be identified, and the most cited publications determined. Methods: A search was performed in the Web of Science (WoS) database using the following keywords: “cytokine”, “inflammatory”, and “eye disease” for the period from 1990 to October 2021. The Citation Network Explorer and the CiteSpace software were then used to analyze the different publications. Results: 3127 publications with 8955 citations generated on the web were found. The largest number of publications on this topic emerged in 2018 and the authors with the largest number of publications addressing this area of research were Peizeng Yang (1.4%), Aize Kijlstra (1.3%), and Stephen C. Pflugfelder (1.2%). Conclusions: the citation network has provided a comprehensive and objective analysis of the main studies on the influence of cytokines in ocular inflammatory diseases.

## 1. Introduction

Eye inflammation is a common clinical problem, encompassing all parts of the eye. Thanks to numerous studies conducted on both human and animal subjects, a more consistent view of the role played by cytokines in inflammatory eye disease has emerged. The presence of cytokines (interferon gamma, IL-2) in ocular tissue obtained from patients with intraocular inflammation (uveitis) has been confirmed. Likewise, cytokines have been shown to induce inflammation in experimental animal models following intraocular injection (IL-l, IL-6, IL-8, tumor necrosis factor (TNF), and granulocyte macrophage-colony stimulating factor (GM-CSF)). Several features that are unique to the eye’s immunology, such as immunosuppression which is associated with anterior chamber-associated immune deviation (ACAID), could be the result of the effects of cytokines. Along this same line, common complications of ocular inflammation, including retinal (macular) edema, keratic precipitates, and neovascularization may be interceded by cytokines. By obtaining a full understanding of the role of cytokines in the development of inflammatory eye disease, it will be possible to lay the foundations for therapies that can revoke the effects of these significant mediators of the inflammatory response [1].

Due to its extreme vascularity, the uvea, the middle layer of the eye, takes the brunt of most serious ocular inflammations. Uveitis comprises a complex group of diseases, which result from diverse etiologies and pathogenic mechanisms. Studies into this group of diseases have provided increasing insight into the role played by cells and biologically active molecules in the immunopathogenesis of these diseases. Uveitis is one of the leading causes of visual impairment and blindness in most countries. However, uveitis remains an idiopathic disease in most cases despite extensive investigation [2]. Recently, our knowledge of the pathogenesis of ocular inflammation has been increased by evidence from studies into human uveitis and animal models, including experimental autoimmune uveitis (EAU), induced by several different retinal antigens, and endotoxin-induced uveitis (EIU), induced by lipopolysaccharide (LPS). There is considerable experimental evidence that suggests the major role that T cells and cytokines play in the pathogenesis of uveitis [3]. Interleukin 2 (IL-2) has been detected in inflamed uveal and retinal tissue, and IL-2 receptor levels are also increased in patients with certain types of uveitis [4]. Finally, the dramatic suppressive effect of cyclosporin-A in controlling uveitis has been repeatedly demonstrated in both EAU and human disease [5]. This immunosuppressive drug inhibits the synthesis of several cytokines, particularly IL-2 and interferon (IFN) gamma. Evidence of the role played by cytokines in the pathogenesis of uveitis originates predominantly from (a) the detection of cytokines in ocular tissue or fluid, (b) in vivo studies in experimental animals, and (c) in vitro studies such as MHC antigen expression.

Tears also play an essential role in maintaining corneal and conjunctival integrity. They provide a tightly regulated, optimal extracellular environment, which proves critical to its numerous functions, including anti-microbial defense, wound healing, and inflammatory responses such as allergies. Elevated levels of inflammatory cytokines have also been reported in tears from various ocular disease states [6].

Ocular allergy and dry eye diseases (DED) are two common, relevant, symptomatic, and not mutually exclusive conditions that affect the ocular surface. These two conditions share certain clinical and biochemical features. Recent advances in our understanding of the pathogenesis of ocular allergy and DED have made it possible to identify several pathways of interaction between these two conditions. A growing body of evidence supports the role of ocular allergy as a risk factor for DED. Ocular allergy, particularly severe forms of keratoconjunctivitis, can have an impact on different key mechanisms of the DED vicious cycle, including tear film instability, ocular surface inflammation, cytokines secretion, and neurosensory abnormalities [7].

By using citation network analysis, it is possible to search for scientific literature on a specific subject. This means that a single publication can provide links to additional and relevant publications, and through the creation of groups, it is also possible to prove, both qualitatively and quantitatively, the relationship that exists between the articles and authors [8]. Furthermore, it is also possible to determine the most cited publication in each group or developed on a field of research or to concentrate the search on a specific topic [9,10].

Considering the increasing number of publications that address cytokines, and the role they play in the development of different inflammatory eye diseases, this study aimed to determine the different fields of research, as well as to identify which was the most cited publication. In addition, the relationships between both the different publications and the research group were analyzed using CitNetExplorer software 1.0.0 (Ness Jan van Eck and Ludo Waltman, Centre for Science and Technology Studies (CWTS), Leiden University, Leiden, The Netherlands). The main purpose of this software is to analyze how scientific literature is developed within a research field.

## 2. Materials and Methods

### 2.1. Database

The WoS database was used to search for the different publications, once the following keywords: “cytokine”, “inflammatory”, and “eye disease” had been established. These specific keywords were chosen as they were in line with the main study objectives, and they are also the most used words across all the fields of research.

Given that there were common articles amongst the results obtained, the Boolean operators NOT and AND, and the “*” characters were used to search for the singular and plural forms of the words. As a result, the words used in the first search were (“cytokine*” AND “inflammat*” AND “ocular” AND “disease*”), the words used in the second search were (“cytokine*” AND “inflammat*” AND “eye” AND “disease*” NOT “ocular”), and the words used in the third search were (“cytokine*” AND “inflammat*” AND “eye*” AND “pathology*” NOT “ocular” NOT “disease*”). The search covered the period from 1990 to October 2021.

While conducting bibliographic searches, whether directly in external databases or in library catalogs, the WoS offers the possibility for these references to be added to the researcher’s library.

With regards to the citation index, the following tools were used in this study: the Social Sciences Citation Index, the Science Citation Index Expanded, and the Emerging Sources Citation Index.

On the other hand, given that authors and organizations use different citation methods, the data was standardized using the CiteSpace software 1.0.0 (Ness Jan van Eck and Ludo Waltman, Centre for Science and Technology Studies (CWTS)). The data was searched and downloaded on the 10th of October 2021.

### 2.2. Data Analysis

The publications were first analyzed using the Citation Network Explorer software 1.0.0 (Ness Jan van Eck and Ludo Waltman, Centre for Science and Technology Studies (CWTS)). This software is used to analyze and visualize the citation networks of scientific publications. Citation networks can be downloaded directly from the WoS, and it is also possible to manage different citation networks containing thousands of inter-related publications and citations. As a result, researchers can use a citation network that contains thousands of publications as the basis for their study, before moving on to focus only on the most relevant ones. By doing so, they can create a small subnetwork of 100 publications, which all cover the same topic.

By using citation score metrics, it was possible to conduct a quantitative analysis of the most commonly cited publications within a specific time interval. This allowed for the quantification of both internal connections that exist within the WoS database and external connections, in which other databases were considered [9].

The CitNetExplorer software 1.0.0 (Ness Jan van Eck and Ludo Waltman, Centre for Science and Technology Studies (CWTS)), is comprised of several techniques used to analyze the different citation networks. The following formula, which was developed by Van Eck in 2012, was used to achieve the clustering function [10].

$$V\left(c_{1\;},\ldots ,c_{n}\right)=\underset{i<j}{\sum }\delta \;\left(c_{i},c_{j}\right)\left(s_{ij}-\gamma \right)$$

The clustering function is used to assign each publication to a group, so the most inter-related publications tended to be found in the same group based on the citation networks [10].

Finally, the identifying core publication’s function was used to analyze the main publications. This function is used to identify the publications considered as the core of a citation network (publications with a minimum number of connections with other core publications). This makes it possible for irrelevant publications to be eliminated. The number of connections is established by the researchers, which means that the higher the value of this parameter, the lower the number of core publications [10]. In this study, publications with 4 or more citations were included in the citation network.

Additionally, the drilling down function was also used given that it allows for a deeper analysis of each group at different levels.

Furthermore, the CiteSpace software 5.6.R2 (Chaomei Chen, College of Computing and Informatics, Drexel University, Philadelphia, PA, USA) was used to perform scientometric analysis. In the scientometric analysis process, certain parameter indicators were also used to carry out a specific assessment: The H-Index (number of times articles were cited in a journal), the degree (number of connections), the centrality (importance of nodes within a research collaboration network), and the half-life (continuity of an investigation from a time perspective) [11,12,13].

## 3. Results

The first articles on the influence of cytokines at the ocular level were published in 1990; therefore, the selected search period was from 1990 to October 2021. Following the WoS search, 3127 publications were found according to title, abstract, and keywords, as well as 8955 citation networks.

**Figure 1:**
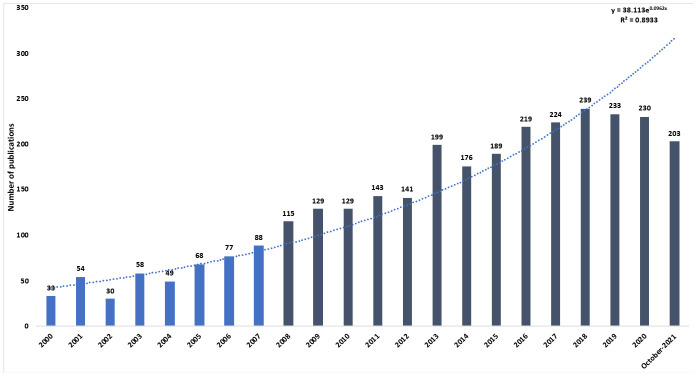
Number of publications per year.

As shown in Figure 1, the number of publications on the influence of cytokines at the ocular level has increased exponentially since 2008 (1990–2007: 20.5%; 2008–October 2021: 79.5%). The highest number of publications emerged in 2018: a total of 239 publications, and 17 citation networks.

### 3.1. Description of the Publications

Of all publications, 87.2% of them were articles, 11.7% were reviews, and 2.9% were proceedings papers.

#### 3.1.1. Language and Countries

**Figure 2:**
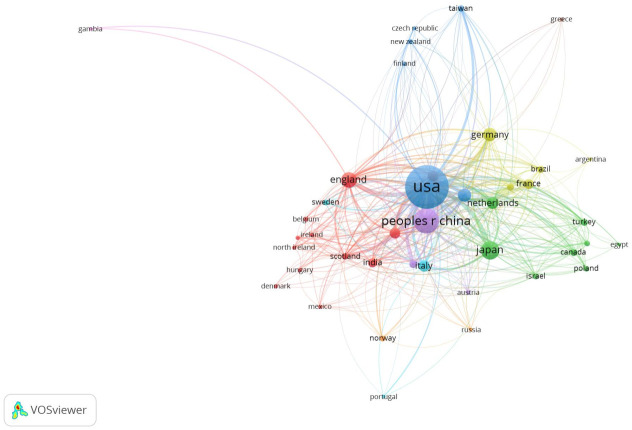
Collaboration between countries.

With regard to the language of the publications, 98.5% were in English, 0.6% were in French, and 0.5% were in German. As shown in Figure 2 and Table 1, the United States (43.2%), China (13.7%), and Japan (8.8%) were the countries with the highest number of publications. Figure 2 shows the most important publications, those publications with the highest number of citations, and it also indicates the group to which each publication belongs. An article’s color represents the group that it belongs to, and the lines between the elements represent the existing links.

Table 1 shows the main characteristics of the four most important groups in Figure 2.

#### 3.1.2. Research Areas

Research on this topic is multidisciplinary. However, the fields of ophthalmology (37.2%) and immunology (11.1%) (Table 2) are particularly worthy of mention.

#### 3.1.3. Authors and Institutions

As shown in Table 3, the authors with the highest number of publications addressing the influence of cytokines at the ocular level were Peizeng Yang (1.4%), Aize Kijlstra (1.3%), and Stephen C. Pflugfelder (1.2%).

Among the institutions with the highest number of publications (Table 4) were Northeastern Illinois University (5.3%), Harvard University (3.5%), and Sun Yat-sen University (2.6%).

#### 3.1.4. Journals

Table 5 shows the journals publishing on the influence of cytokines at an ocular level, and the number of said publications according to the WoS database. The Scimago Journal Rank (SJR) quartile has also been included in the table, emphasizing the importance and relevance of the journals with the largest amount of content published on this topic.

#### 3.1.5. Keywords

**Figure 3:**
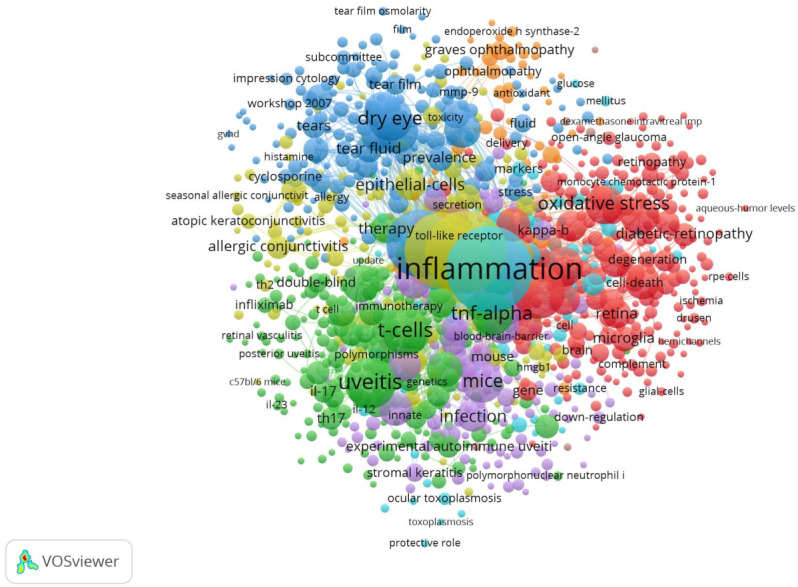
Link between keywords.

On the other hand, the most used keywords were “inflammation” (127 publications), “cytokine” (125 publications), and “dry eye disease” (118 publications). Table 6 and Figure 3 show the most used keywords in the most relevant publications and the frequency of their appearance in publications with other keywords.

Table 7 shows the main characteristics of the 5 most important groups in Figure 3.

### 3.2. Most Cited Publications

The most cited publication was the article written by Liddelow et al. [14], which was published in 1999 with a citation index of 2166. In this study, the authors showed a reactive astrocyte subtype (A1) induced by activated neuroinflammatory microglia. Active microglia produce A1 astrocytes while secreting Il-1α, TNF, and C1q. A1 astrocytes lose the ability to promote neuronal survival, growth, synaptogenesis, and phagocytosis, and induce neuron and oligodendrocyte death. In turn, by blocking the formation of A1 astrocytes, the death of axotomized central nervous system (CNS) neurons in vivo is prevented. Overall, these results helped explain why CNS neurons die after axotomy.

Within the 20 most cited articles, 15 of them discussed the relevance of the cytokines in the tear fluid of patients with dry eye disease. In turn, two of them attempted to identify the presence of cytokines in ocular allergies (Table 8), and three of them analyzed the presence of cytokines in uveitis.

### 3.3. Clustering

The clustering function was used to assign each publication into a group, so publications that were close to each other within the citation network were in the same group. This means that each group was comprised of strongly linked publications in terms of their citation connections. As such, it was possible to interpret that each group represents a specific topic within the scientific literature. To distinguish between the groups, each group was assigned a different color and the connections between the groups are shown using colored lines.

Nine groups were identified in this analysis, five of them containing a significant number of publications. The four remaining groups only represented 3.4% of all the publications on cytokines and ocular inflammatory diseases.

#### 3.3.1. Cluster Group 1

In Group 1, 765 publications and 3758 citations were found throughout the whole network. The most cited publication was the article by Solomon et al. [21], which was published in JAMA Ophthalmology in 2001. The objective of this study was to compare the expression of the pro- and anti-inflammatory forms of interleukin (IL)-1 in the tear fluid and conjunctival epithelium in normal eyes and those with dry eye disease. The concentrations of IL-1a, IL-1b (precursor and mature forms), and IL-1 receptor antagonist (IL-1Ra) were measured using ELISA in tear fluid samples obtained from normal individuals and from patients with dry eye with rosacea-associated meibomian gland disease (MGD) and Sjogren’s syndrome (SS) or aqueous tear deficiency (ATD). The study suggested that dry eye disease is accompanied by an increase in the pro-inflammatory forms of IL-1 (IL-1a and mature IL-1b) and a decrease in the biologically inactive precursor IL-1b in tear fluid. The conjunctival epithelium appears to be one source of the increased concentration of IL-1 in the tear fluid of patients with dry eye disease. These results suggested that IL-1 may play a key role in the pathogenesis of keratoconjunctivitis sicca.

**Figure 4:**
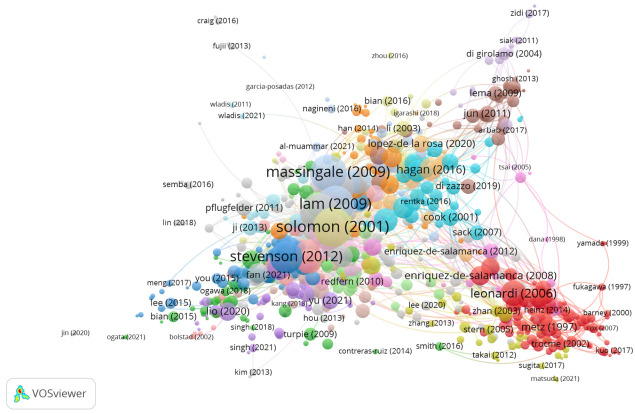
Citation network in Group 1.

The articles in this group addressed the relevance of cytokines in the tear fluid of patients suffering from dry eye (Figure 4).

#### 3.3.2. Cluster Group 2

In Group 2, 611 publications and 1829 citations were found across the whole network. The most cited publication was the article by Amadi-Obi et al. [16], which was published in *Nature Medicine* in 2007. In this study, the authors showed the participation of T helper type 17 cells (TH17) in uveitis and scleritis. In addition, they provided validation of a uveitis model, experimental autoimmune uveoretinitis (EUA). Thus, the authors concluded that TH1 cells can reduce uveitis by antagonizing the TH17 phenotype through the induction of interleukin IL-27 mediated by IFN-γ in the target tissue. On the other hand, interleukin IL-2 favors the expansion of TH17, which helps to explain the efficacy of IL-2R antibody therapy in uveitis. Likewise, it suggests that the antagonism of TH17 by IFN-γ and/or IL-27 could be used for the treatment of chronic inflammation.

**Figure 5:**
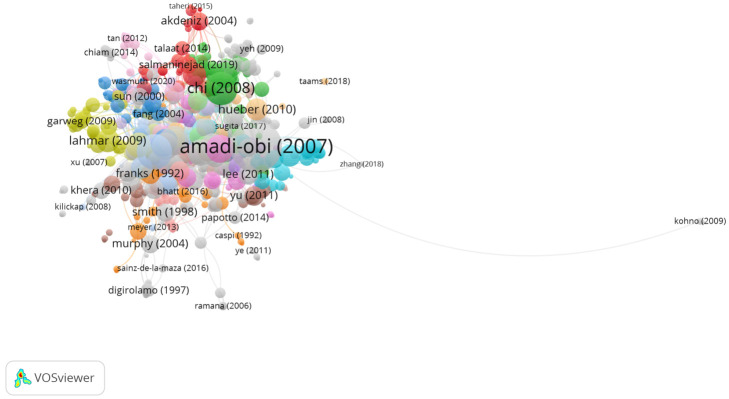
Citation network in Group 2.

Therefore, the articles in this group analyzed the presence of cytokines and chemokines in uveitis (Figure 5).

#### 3.3.3. Cluster Group 3

In Group 3, 534 publications and 1252 citations were found across the whole network. The most cited publication was the article by Xu et al. [27], which was published in *Progress in Retinal and Eye Research* in 2009. This study showed how para-inflammation is also present in aging retinas and can contribute to age-related retinal pathologies. Thus, at the retinal/choroidal interface, the activation of complement activity in Bruch’s membrane, retinal pigment epithelial cells, and the accumulation of microglia in the subretinal space show para-inflammation. With age, para-inflammatory changes were also observed in the choroidal tissue, evidenced by an increase in the thickness of the choroid, a greater number of CD45 + CRIg + macrophages, morphological abnormalities in choroidal melanocytes, and fibrosis in the choroidal tissue.

**Figure 6:**
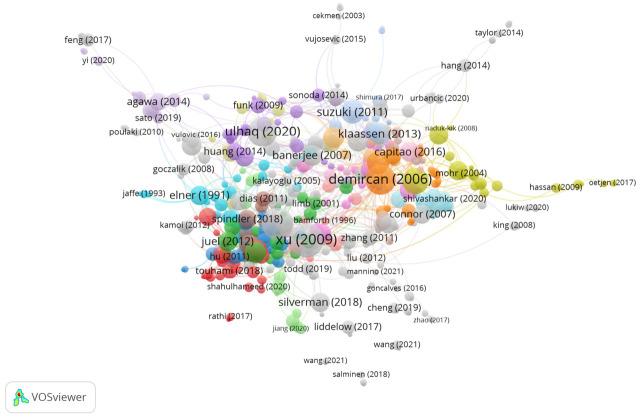
Citation network in Group 3.

The publications in this group aimed to analyze intraocular cytokine levels and the prevalence of intraocular antiretinal antibodies in patients with retinitis pigmentosa, age-related macular degeneration, uveitis, glaucoma, and cataracts, before going on to correlate the results with the clinical manifestations (Figure 6).

#### 3.3.4. Cluster Group 4

In Group 4, 254 publications and 751 citations were found across the whole network. The most cited publication was the article by Hazlett et al. [34], which was published in *Progress in Retinal and Eye Research* in 2004. In this study, it was demonstrated that, in pseudomonas aeruginosa, the sustained production of IFN gamma driven by IL-12 in dominant Th1-sensitive strains, such as C57BL/6 (B6), contributed to the destruction and perforation of the cornea, while IL-18 driven IFN gamma production in the absence of IL-12 was associated with bacterial death and less corneal destruction in Th2-responsive dominant strains, such as BALB/c.

**Figure 7:**
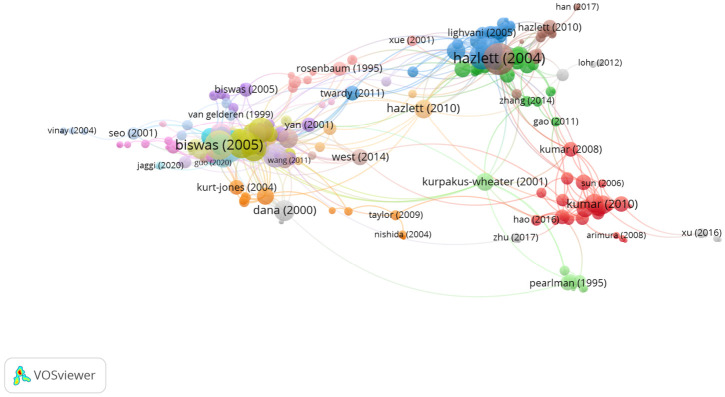
Citation network in Group 4.

The articles in this group analyzed the role of cytokines in anterior segment pathologies (Figure 7).

#### 3.3.5. Cluster Group 5

In Group 5, 124 publications and 353 citations were found across the whole network. The most cited publication was the article by Wang et al. [35], which was published in *Cell* *Biology and Metabolism* in 1996. In this study, the authors demonstrated that leukoregulin, a 50 kDa product of activated T lymphocytes, dramatically increased PGE2 synthesis in cultured human orbital fibroblasts and increased PGHS-2 expression in dermal fibroblasts. Orbital and dermal fibroblasts expressed high levels of mRNA and protein of PGHS-1, the other abundant form of cyclooxygenase. On the other hand, unlike the expression of PGHS-2, leukoregulin did not alter the levels of PGHS-1 in orbital or dermal fibroblasts, suggesting that PGHS-1 does not participate in cytokine-dependent prostanoid production in human fibroblasts. These findings clarified the relevant pathogenetic mechanism for the intense inflammation associated with Graves’ ophthalmopathy.

**Figure 8:**
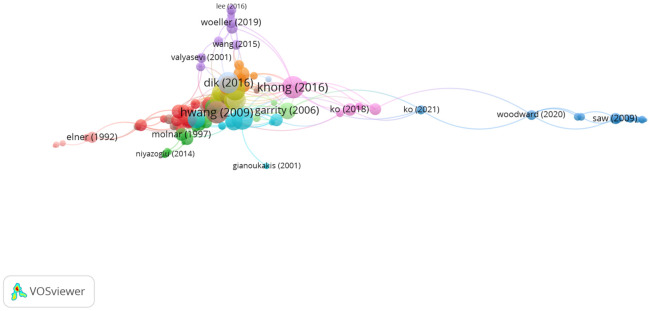
Citation network in Group 5.

Therefore, the articles in this group analyzed the influence of cytokines and chemokines in Graves’ disease (Figure 8).

### 3.4. Core Function

**Figure 9:**
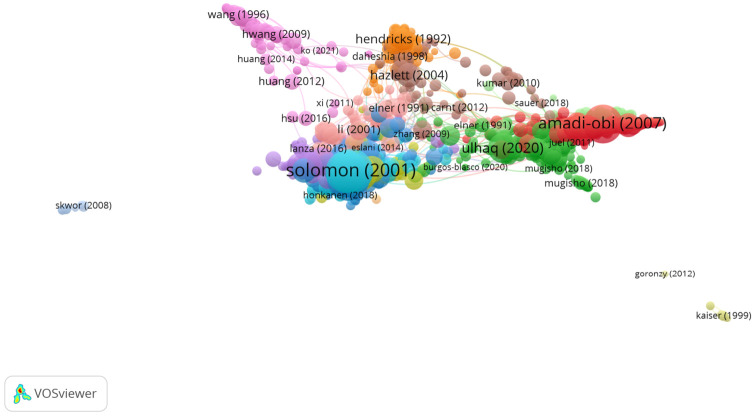
Core publications on the cytokines and ocular inflammatory diseases citation network.

A total of 1299 publications with four or more citations were found, and the citation network was 6732, representing 40.4% (Figure 9). Therefore, the subject of focus is multidisciplinary. Although the main topic identified was the relevance of cytokines and chemokines in the tears of patients with dry eye disease.

## 4. Discussion

Bibliometric studies are quantitative analysis methods. They use mathematical and statistical tools to measure the interrelationships and impacts of publications within a specific area of research. These methods allow an overview of large amounts of academic literature and can be used to identify influential studies, authors, journals, organizations, and countries over time. Moreover, through citation network analysis, the different research areas can be identified more intuitively using mapping social networks that include co-word, co-authorship, and co-citation analysis. It also allows examining the relationships between research groups, institutions, and even countries. In general, the citation network analysis provides a clear diagram of the most cited publications in a research area [36]. The analysis of the influence of cytokines on inflammatory eye diseases could help many researchers to identify collaboration opportunities among colleagues, multidisciplinary possibilities of teamwork, and to create new research groups with an international vision [37].

The main databases, such as Scopus or WoS, allow citation networks to be created. However, their use is limited when conducting a systematic review of all the existing literature on a subject, given that they do not provide a general overview of the existing connections between the citations of a group and their publications. For this reason, CitNetExplorer software 1.0.0 (Ness Jan van Eck and Ludo Waltman, Centre for Science and Technology Studies (CWTS)) and CiteSpace software 5.6.R2 (Chaomei Chen, College of Computing and Informatics, Drexel University, Philadelphia, PA, USA) were used to visualize, analyze, and explore the citation networks of scientific publications, as they provide a more in-depth analysis than databases such as WoS or Scopus when creating citation networks [10].

The main objective of this study was to analyze the existing literature about the influence that cytokines have on inflammatory eye diseases. To do so the WoS database was used. With a search range dating back to 1900, it is one of the most extensive databases. Nonetheless, it should be noted that the WoS only accepts international journals that have passed a scrupulous selection process.

Therefore, once the existing bibliography had been downloaded from the WoS, it was possible to use the CitNetExplorer software 1.0.0 (Ness Jan van Eck and Ludo Waltman, Centre for Science and Technology Studies (CWTS)) and CiteSpace software 5.6.R2 (Chaomei Chen, College of Computing and Informatics, Drexel University, Philadelphia, PA, USA) to collect and analyze all the available literature on the impact of cytokines on ocular disease. By performing citation network analysis, it was also possible to assess the links between the fields of study and the different research groups, using the clustering function. This function allows for publications to be grouped according to the connections that exist between the citations. The drilling down function was also used to conduct a more in-depth analysis of each group’s bibliography. The core publication’s function identifies the main publications, that is to say, those with a minimum number of citations (more than 4 citations). Therefore, by using these functions, it was possible to completely analyze and study the research that has been done on the specific field of research. The methodology was based on previous citation network studies that have been conducted recently in other fields of study [38,39,40,41,42].

One of the first publications on cytokines in eye diseases was published by Rosenbaum et al. [43] in 1991. This study tested the activity of a soluble human interleukin-1 receptor injected intravitreally in a rabbit model of IL-1.3 ug-induced inflammation of the soluble receptor and completely inhibited cellular infiltration and protein extravasation within 6 h after an intravitreal injection of 10.5 ng of recombinant human interleukin-1 alpha. The soluble receptor efficacy was less marked 24 h after the IL-1 injection. The cellular infiltrate did not decrease when the IL-1 receptor was injected 2 h after IL-1. Therefore, the conclusion was that a study on receptor activity would be necessary as a therapeutic modality for inflammatory eye disease. It was not until 2008 that there was an exponential increase in the number of publications on the influence of cytokines at the ocular level. This was due to increasing research on inflammatory mediators in the tear, for example, the publications by Lam et al. [33] and Massingale et al. [44] in 2009. Both studies concluded that dry eye patients had significantly higher concentrations of cytokines in their tears, which correlated with the severity of the disease. In fact, the highest number of publications emerged in 2020, coinciding with a significant increase in studies into immunology and, specifically, cytokines, including the side effects of COVID-19. From 2020 is the article by Alam et al. [45], which concluded that a reduction in goblet cell density correlated with more severe conjunctival disease, greater IFN-γ expression, and the maturation of antigen-presenting cells stood out. Thus, the evidence suggested that dry eye therapies that suppress IFN-γ expression preserve the number and function of conjunctival goblet cells and should be considered in aqueous deficiency.

The countries with the highest number of publications were the United States, China, and Japan. This may be related to the fact that fungal keratitis is one of the main causes of cough in the United States and industrialized countries. For this reason, countries with a higher income and therefore better infrastructures are currently working on this research topic, resulting in a higher number of publications. Similarly, the upward trend in the number of publications from countries such as the United States or Great Britain has been linked to a range of factors, for example, the fact that these are English-speaking countries and the possible affiliations that may exist between the different research groups within the scientific community [46,47]. In turn, our studies concurred with other bibliometric studies, where China and the USA were the countries with the highest number of publications [48,49]. Furthermore, in the study carried out by Shanin et al. [48], the collaboration between the USA and South Korea was observed in the citation network analysis, highlighting the importance of analyzing the connections between publications, given that we believe that in the coming years, there will be a significant increase in the number of publications emerging from South Korea.

At the same time, and according to other bibliometric studies, Harvard University was among the institutions with the most publications. This once again confirmed the importance of this research field in the United States.

However, in our study, Enriquez de Salamanca, the author with the highest number of publications, is Spanish, emphasizing the importance of highlighting the increased interest in this field of research by Spanish researchers, often working in collaboration with researchers from other countries.

Regarding journals with a high number of publications, *Investigative Ophthalmology & Visual Science* stood out. This journal ranked tenth in the ophthalmology category with an impact factor of 3.47. This was because this open access journal had the highest number of citations and publications in the field of ophthalmology in recent years. In addition, the journal with the highest impact factor was *Frontiers in Immunology*, 7.56. However, it is worth considering that although the impact factor is a critical index of a journal’s importance, it is not an absolute measure index. The main difference between these indices is that the latter is based on the impact of the research results, in addition to the intellectual and physical contributions made by the authors [10].

Regarding the years, despite the significant increase in the number of publications in recent years, the publications with the greatest impact and with the highest number of citations were published before 2010. Among the top 20 publications, the one published by Wang et al. [26] showed that IL-35 induced Breg cells and promoted their conversion to a Breg subset that produced IL-35 as well as IL-10. Furthermore, IL-35 treatment in mice was shown to protect against experimental autoimmune uveitis (UAE). Mice lacking IL-35 or defective in IL-35 signaling produced fewer Breg cells endogenously and eventually developed severe uveitis. Therefore, the authors concluded that IL-35 can treat autoimmune and inflammatory diseases. In 2017, the article published by Pflugfelder et al. [50] was highlighted in 443 citations. The authors studied how cyclosporine and lifitegrast, the two therapies approved by the US Food and Drug Administration, inhibited T cell activation and cytokine production. These therapies represent an important advance in dry eye therapy but are not effective in improving discomfort and disease of the corneal epithelium in all patients. Another treatment for dry eye is Tacrolimus 0.03% eyedrops and ointments. This treatment reduces inflammatory cells and cytokines on the ocular surfaces. As the dry eye is a chronic disease and requires long-term treatment, therapies that target the main pathophysiological conditions without many adverse effects are required. Thus, in vitro assays proved that lifitegrast inhibited the release of cytokines, interferon gamma, tumor necrosis factor alpha (TNF-α), and other interleukins (ILs) [51,52]. In the treatment of uveitis, sulfasalazine is prescribed for anterior uveitis and azathioprine for posterior uveitis. Although azathioprine and infliximab are also prescribed, methotrexate remains the most widely drug used as a second immunomodulator [53]. Regarding retinal pathologies, vascular endothelial growth factor (VEGF) is the most studied molecule that can increase vascular permeability. Regular intravitreal injections of anti-VEGF agents can improve vision and reduce the accumulation of macular fluid in age-related macular degeneration. This therapy is the main treatment modality for age-related macular degeneration; however, clinical trials have shown that ~40% of patients are resistant to anti-VEGF. To identify a new target molecule or a new biomarker to predict anti-VEGF resistance, the relationship between the response to anti-VEGF treatment and the concentration of intraocular inflammatory cytokines/chemokines was examined. Thus, in the study by Shimura et al. [54], a favorable response was obtained in patients with elevated basal aqueous VEGF, soluble VEGF receptor 1, monocyte chemoattractant protein 1 (MCP-1), ICAM-1, IL-6, and IP-10. Felfeli et al. [55] compared aqueous cytokine concentrations at baseline and two months after anti-VEGF therapy and reported that the aqueous levels of ICAM-1, MCP-1, placental growth factor, and TGF-β2 were significantly decreased in patients with a favorable response. Graves’ disease is a condition caused by an autoimmune process that affects the thyroid gland, the main result of which is hyperthyroidism. Therefore, these new discoveries led to the investigation of promising therapies, such as immunotherapies against specific antigens (for example, against thyroid-stimulating-hormone receptor), to restore immune tolerance against the dominant immune epitopes associated with the autoimmunity in Graves’ disease. Other treatments, etanercept (which blocks tumor necrosis factor-mediated inflammatory responses), tocilizumab (TCZ) (which acts against the IL-6 receptor), and rituximab (which acts against CD20), are useful and safe therapeutic options. Additionally, teprotumumab (a human monoclonal anti-IGF-1R blocking antibody) is effective in treating patients with moderate to severe disease. Finally, molecules capable of acting as CXCR3 antagonists or blocking CXCL10 are also being studied [56].

One of the most relevant studies was that carried out by Solomon et al., in which the authors investigated the pro- and anti-inflammatory forms of interleukin-1 in the tear fluid and conjunctiva of patients with dry eye disease. They demonstrated that the conjunctival epithelium appeared to be one source of the increased concentration of IL-1 in the tear fluid of patients with dry eye disease and that IL-1 played a key role in the pathogenesis of keratoconjunctivitis sicca. It is important to note that two subsequent articles by Massingale et al. [44] and by Enriquez-de-Salamanca et al. [57] also supported these results, once again highlighting the role of IL-1 as a key player in DED. Besides, five new inflammatory cytokines were elevated in patients with dry eye disease: fractalkine, IL-1Ra, IL-6, IL-8/CXCL8, and EGF levels and were correlated with pain and with clinical parameters measuring tear stability and tear production or ocular surface integrity. All these articles, found in Group 1 of our citation network, suggest that inflammation plays an important role in the development of severe DED.

Regarding the influence of cytokines on ocular allergies, one of the reference studies was published by Cook et al. in 2001 [6]. The authors attempted to identify the presence of cytokines on ocular allergies. Tears were collected from the inferior fornix of seven non-allergic and naïve allergic donors, which were subsequently analyzed using flow cytometry. They found that all six cytokines were detectable in both non-allergic and allergic tears, but that tears from allergic donors contained significantly less IL-10, and there were significant increases in the ratios of TNFalpha/IFNgamma, IL-5/IFNgamma and IL-5/IL-10. In fact, the relation between allergies and inflammatory cytokines has been studied extensively and publications have been aggregated in Group 2 of our citation network. One relevant article on this topic was the publication by Leonardi et al. in which authors analyzed the presence of cytokines in the tears of subjects with allergic conjunctivitis [58]. The results showed that IL-1β, IL-2, IL-5, IL-6, IL-12, IL-13, and MCP-1 increased in all the tears of allergic groups compared to controls. In addition, IL-4, IFN-γ, and IL-10 were elevated in active seasonal allergic conjunctivitis and vernal keratoconjunctivitis. By analyzing all the publications in this group of the citation network it was possible to conclude that the difference in cytokine levels in the different patients may suggest that tears are a useful indicator of the immune mechanisms that occur during allergic conjunctivitis.

Another relevant study was published by Wakefield et al. in 1992 in *Cytokine* [1], which demonstrated the presence of cytokines (interferon gamma, IL-2) in the ocular tissue of patients with intraocular inflammation (uveitis). This was the most cited publication in Group 3 of our citation network. The researchers demonstrated that inflammation could be induced in experimental animals following the intraocular injection of several cytokines (IL- 1, IL-6, IL-8, TNF, granulocyte macrophage-colony stimulating factor (GM-CSF)). This article and the other publications included in the group suggest that common complications of ocular inflammation such as glaucoma, uveitis, retinitis, keratic precipitates, retinal (macular) edema, and neovascularization may be mediated by cytokines.

Finally, the last group, Group 5 of our citation network, analyzed the influence of cytokines and chemokines in Graves’ disease. The article by Metcalfe et al. published in *Clinical Endocrinology* [59] in 1994, is particularly worth mentioning. In this article, the authors demonstrated that extraocular muscle fibroblasts are more sensitive than dermal fibroblasts to cell-derived cytokines, which may partly explain the anatomical location of the ophthalmopathy.

## 5. Conclusions

In conclusion, this study offers a comprehensive and objective analysis of the main papers on the influence of cytokines on inflammatory eye diseases. In this study, five main groups were found on the influence of cytokines on inflammatory eye diseases (DED, allergies, inflammatory ocular diseases, anterior segment, and Graves’ disease). Cytokines in tears was the most researched topic. Furthermore, by using the WoS database and the Citation Network Explorer software 1.0.0 (Ness Jan van Eck and Ludo Waltman, Centre for Science and Technology Studies (CWTS)), it was possible to visualize, analyze, and explore the most cited articles and citation networks that exist to date.

There has been a growing number of citation network studies because it is the only method of analysis that provides a global overview of the different fields of study on a specific topic. Moreover, the CitNetExplorer software 1.0.0 (Ness Jan van Eck and Ludo Waltman, Centre for Science and Technology Studies (CWTS)) makes analyzing all studies on a topic possible, allowing for more detailed research to be conducted. This might change how research is conducted in different fields of study.

## Figures and Tables

**Table 1:** Characteristics of the main countries.

Group	Color	Main Countries	Publications	Centrality	Degree	Half-Life	Connections
1°	Red	England	112	0.21	33	18.5	604
2°	Green	Japan	152	0.10	15	16.5	506
3°	Blue	USA	875	0.86	60	22.5	2328
4°	Yellow	Germany	85	0.16	31	16.5	230

**Table 2:** The 10 research areas with the highest number of publications.

Category	Frequency	Centrality	Degree	Half-Life
Ophthalmology	668	0.12	14	21.5
Immunology	397	0.37	27	21.5
Biochemistry and Molecular Biology	199	0.32	32	21.5
Cell Biology	179	0.15	22	22.5
Science and Technology-Other Topics	115	0.15	20	22.5
Multidisciplinary Sciences	106	0.00	5	22.5
Research and Experimental Medicine	90	0.08	22	24.5
Pharmacology and Pharmacy	86	0.15	19	23.5
Neurosciences and Neurology	73	0.11	16	17.5
Pathology	46	0.03	12	21.5

**Table 3:** The 10 authors with the highest number of publications.

Author	Number of Publications	*H* Index	Total Citations	Citation Average	Degree	Connections
Peizeng Yang	38	6	166	15.09	22	181
Aize Kijlstra	37	7	180	16.36	19	178
Stephen C. Pflugfelder	31	27	3168	83.37	15	417
Cintia S. De Paiva	27	17	1086	47.22	13	362
Charles E. Egwuagu	23	8	173	19.22	14	222
Amalia Enriquez de Salamanca	18	12	552	32.47	9	425
Margarita Calonge	17	29	2575	21.46	10	840
C.C. Chan	17	18	837	34.88	21	130
Michael E. Stern	17	18	1895	75.80	18	477
Reza Dana	17	9	289	26.27	6	174

**Table 4:** The 10 institutions with the highest number of publications.

Category	Frequency	Centrality	Degree	Half-Life	Connections
Northeastern Illinois University	96	0.00	39	20.5	473
Harvard University	63	0.00	38	13.5	322
Sun Yat-sen University	46	0.00	12	4.5	213
Baylor College of Medicine	41	0.00	15	10.5	443
Chongqing Medical University	33	0.00	8	5.5	149
Johns Hopkins University	30	0.00	27	14.5	131
Fudan University	27	0.00	9	9.5	114
Case Western Reserve University	26	0.00	18	15.5	35
University of California, Los Angeles	22	0.00	10	5.5	168
Boston University	22	0.00	50	10.5	70

**Table 5:** The 10 journals with the highest number of publications.

Journal	Total Publications	Impact Factor (2020)	Quartile Score	SJR (2020)	Citations/Docs (2 Years)	Total Citations (2020)	*H* Index	Country
Investigative Ophthalmology & visual science	301	4.80	Q1	1.93	4.045	8469	218	United States
Experimental eye research	109	3.47	Q2	1.22	3.161	2740	125	United States
Plos One	100	3.24	Q2	0.99	3.041	185,483	332	United States
Journal of immunology	79	5.42	Q2	2.74	4.609	12,042	372	United States
Ocular immunology and inflammation	58	3.07	Q2	0.77	1.938	1292	56	United Kingdom
Frontiers in Immunology	54	7.56	Q1	2.65	6.756	54,184	124	Switzerland
Current eye research	51	2.42	Q3	0.83	2.146	1438	81	United Kingdom
Scientific reports	51	4.38	Q1	1.24	4.130	282,734	213	United Kingdom
Molecular vision	50	2.37	Q3	0.89	2.228	685	92	United States
Cornea	44	2.65	Q3	1.27	1.942	2121	117	United States
International Journal of Molecular Sciences	43	5.92	Q1	1.45	5.542	77,153	162	Switzerland

**Table 6:** The 30 most used keywords.

Keyword	Frequency	Degree	Total Link Strength
Cytokine	833	122	4837
Inflammation	769	109	6215
Expression	679	100	5408
Disease	489	93	3553
Cell	288	102	2053
T cell	282	104	1740
Activation	279	84	2126
Uveitis	262	97	2212
Dry eye	208	82	1740
Gene expression	190	89	1341
Ocular surface	184	67	1487
TNF alpha	168	74	1479
Pathogenesis	154	63	1262
Endothelial growth factor	147	55	1258
Necrosis factor alpha	145	69	1062
Rheumatoid arthritis	144	65	1024
Dry eye disease	144	54	1162
Receptor	140	65	930
Oxidative stress	135	53	1103
Mechanism	133	58	936

**Table 7:** Characteristics of most used keywords.

Cluster	Color	Main Keywords	Topic	%
1	Red	Activation, apoptosis, oxidative stress, macular degeneration, diabetic retinopathy	Relevance of cytokines in retinal pathologies	22.5
2	Green	Uveitis, induction, t cells, rheumatoid-arthritis, TNF-alpha	Relevance of cytokines in corneal pathologies	20.4
3	Blue	Dry eye, ocular surface, tears, disease, tear fluid	Cytokines and dry eye	16.1
4	Yellow	Expression, vernal conjunctivitis, eye, epithelial cells, allergic conjunctivitis	Cytokines and eye allergies	14.1
5	Purple	Mice, dendritic cells, infection, interferon gamma, endophthalmitis	Cytokines in the anterior segment	13.4

**Table 8:** Description of the 20 most cited publications on cytokines and ocular inflammatory diseases.

Author	Title	Journal	Year	Citation Index	Links
Liddelow et al. [14]	Neurotoxic reactive astrocytes are induced by activated microglia	Nature. 2017 Jan;541(7638):481–487.	2017	2166	3
Hueber et al. [15]	Effects of AIN457, a Fully Human Antibody to Interleukin-17A, on Psoriasis, Rheumatoid Arthritis, and Uveitis	Sci. Transl. Med. 2010 Oct 6;2(52):52ra72.	2010	666	16
Amadi-Obi et al. [16]	TH17 cells contribute to uveitis and scleritis and are expanded by IL-2 and inhibited by IL-27/STAT1	Nat. Med. 2007 Jun;13(6):711–8.	2007	605	80
Stern et al. [17]	The pathology of dry eye: the interaction between the ocular surface and lacrimal gland	Cornea. 1998 Nov;17(6):584–9.	1998	564	45
Sangiovanni et al. [18]	The role of omega-3 long-chain polyunsaturated fatty acids in health and disease of the retina	Prog. Retin. Eye Res. 2005 Jan;24(1):87–138.	2005	529	5
Vasan et al. [19]	Inflammatory Markers and Risk of Heart Failure in Elderly Subjects Without Prior Myocardial Infarction: the Framingham Heart Study	Circulation. 2003 Mar 25;107(11):1486–91	2003	520	20
Baudouin et al. [20]	Preservatives in eyedrops: the good, the bad and the ugly	Prog. Retin. Eye Res. 2010 Jul;29(4):312–34.	2010	515	8
Solomon et al. [21]	Pro- and anti-inflammatory forms of interleukin-1 in the tear fluid and conjunctiva of patients with dry-eye disease	Ophthalmol. Vis. Sci. 2001 Sep;42(10):2283–92	2001	500	111
Connor et al. [22]	Increased dietary intake of ω-3-polyunsaturated fatty acids reduces pathological retinal angiogenesis	Nat. Med. 2007 Jul;13(7):868–873	2007	487	7
Kurt-Jones et al. [23]	Herpes simplex virus 1 interaction with Toll-like receptor 2 contributes to lethal encephalitis	Proc. Natl. Acad. Sci. USA. 2004 Feb 3;101(5):1315–20	2004	457	4
Kern et al. [24]	Contributions of Inflammatory Processes to the Development of the Early Stages of Diabetic Retinopathy	Exp. Diabetes Res. 2007;2007:95103.	2007	450	18
Pflugfelder et al. [25]	Altered cytokine balance in the tear fluid and conjunctiva of patients with Sjögren’s syndrome keratoconjunctivitis sicca	Curr. Eye Res. 1999 Sep;19(3):201–11.	1999	443	108
Wang et al. [26]	Interleukin-35 induces regulatory B cells that suppress autoimmune disease	Nat. Med. 2014 Jun;20(6):633–41.	2014	433	17
Xu et al. [27]	Para-inflammation in the aging retina	Prog. Retin. Eye Res. 2009 Sep;28(5):348–68.	2009	422	28
Luo et al. [28]	Experimental Dry Eye Stimulates Production of Inflammatory Cytokines and MMP-9 and Activates MAPK Signaling Pathways on the Ocular Surface	Invest. Ophthalmol. Vis. Sci. 2004 Dec;45(12):4293–301	2004	396	105
Penfold et al. [29]	Immunological and Aetiological Aspects of Macular Degeneration	Prog. Retin. Eye Res. 2001 May;20(3):385–414	2001	346	11
Klaassen et al. [30]	Molecular basis of the inner blood-retinal barrier and its breakdown in diabetic macular edema and other pathological conditions	Prog. Retin. Eye Res. 2013 May;34:19–48.	2013	338	10
Stevenson et al. [31]	Dry eye disease: an immune-mediated ocular surface disorder	Arch. Ophthalmol. 2012 Jan;130(1):90–100	2012	327	78
Stern et al. [32]	The role of the lacrimal functional unit in the pathophysiology of dry eye	Exp. Eye Res. 2004 Mar;78(3):409–16.	2004	316	27
Lam et al. [33]	Tear cytokine profiles in dysfunctional tear syndrome	Am. J. Ophthalmol. 2009 Feb;147(2):198–205. e1.	2009	310	111

## Data Availability

Not applicable.
